# Biomarkers and testing in pathology

**DOI:** 10.1097/MOU.0000000000001363

**Published:** 2026-01-29

**Authors:** Eva Compérat

**Affiliations:** Department of Pathology, Medical University Vienna, Vienna, Austria

**Keywords:** bladder cancer, *BRCA*, *FGFR*, kidney cancer, prostate cancer, testing

## Abstract

**Purpose of review:**

We try to highlight the most current testing methods in uropathology. Most of these tests are not fully approved, but nevertheless have found their way to daily routine.

**Recent findings:**

In the bladder, testing is mainly based on immunohistochemistry and somatic testing. Other organs such as the prostate, the kidney, and the testis require sometimes different methods.

**Summary:**

Although pathology is still the gold standard in diagnosis of tumor for patients in uropathology, many clinicians want to improve treatment options and try to tailor personalized treatments. This is of course depending not only on the available treatment options, but also on the somatic or germcell background of the patient and his tumor.

## INTRODUCTION

During these last years, translational research has become an important tool to advance treatment strategies in urology. Because of tailored treatment strategies, somatic and genetic testing have moved into the focus of patient's management. In bladder, prostate and testis, these strategies are widely spread and used, in renal cell carcinomas, testing seems to be more complicated, maybe also linked to the numerous subtypes, which exist in kidney kidney tumor diagnostic.

Important progress has been made in urothelial carcinoma, not only with the help of immunohistochemistry but also with genetic testing. In metastatic castration-resistant prostate cancer (mCRPC), testing has also become part of the patient's management, voices are even raising to test patients in earlier stages, such as the metastatic hormone-sensitive setting (mHSPC). In testicular cancer, the evaluation of distinct microRNA profiles in the serum allow clinicians to monitor disease status in these mostly young patients. In renal cell carcinomas (RCC), the detection of histologically sarcomatoid aspects in the tumor and the evaluation of PD-L1 on a immunhistochemical base can also help to guide treatment decision.

Therefore, it is of high importance to know which marker and target therapies exist in order to help clinicians in decision-making as part of the multidisciplinary team. 

**Box 1:**
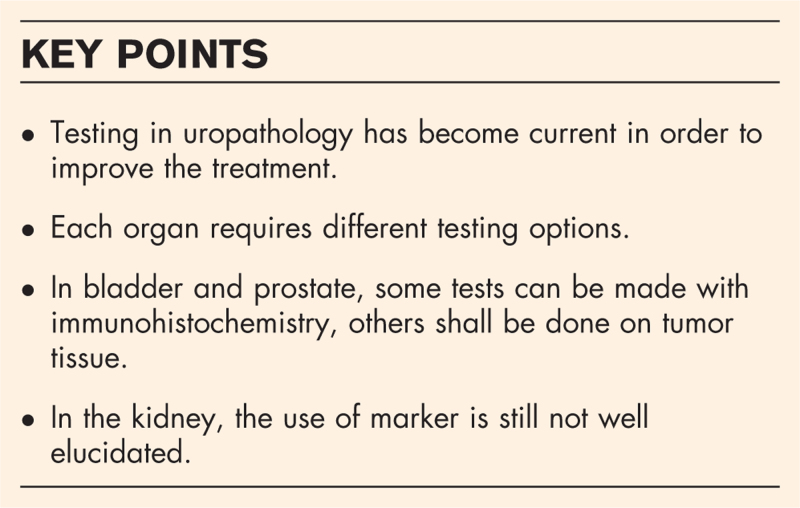
no caption available

## TESTING IN BLADDER

In this article, we will not touch the developing field of liquid biopsies in bladder cancer, as this is mostly not recommended by official pathology societies.

Bladder pathology is complex as urothelial carcinoma is a very heterogeneous disease. Much progress has been made with regards to the exploration of urothelial carcinogenesis, but several steps in the development of urothelial carcinoma are still not a 100% understood.

For many years, researchers, clinicians and pathologists have tried to gain a deeper understanding of urothelial carcinoma on a molecular level. Different molecular classifications of urothelial carcinoma invading the muscle (pT2-4) have been published, a consensus classification found its way in 2020. This consensus classification system is valid for muscle invasive bladder cancer (MIBC) and is based on six different variants of urothelial carcinoma. The suggested groups are: luminal with the subgroups luminal papillary, luminal nonspecified and luminal unstable, furthermore stoma-rich, then basal/squamous and neuroendocrine-like. The published data could show that these different tumor variants have different oncogenic mechanisms and different ‘key’-mutations/genes. The overlap with histology is not 100%, but some overlaps can obviously be observed [1]. Research has shown that some variants react better to specific chemotherapies, and can show different metastatic pattern, but outside clinical trials, the molecular expression is still not recommended nor part of the routine clinical work-up [2,3].

For the purpose of testing and targeted therapies, immunohistochemistry plays an important role, and as already said before, the landscape of treatment has enormously evolved. It is important to realize that many of the marker connected to a targeted therapy are tested based on immunohistochemistry. The most prevalent marker is PD-L1, which has been tested in many trials with regards to immune checkpoint inhibitors (ICI) [4].

Different antibodies and different scoring systems exist: one of the first antibodies was the sp142 antibody (Ventana), testing the PD-L expression in tumor-related immune cells. If the expression of PD-L1 is/was at least 5% in the tumor-related immune cells, the clinician is/was allowed to treat the patient with atezolizumab [5]. Another system of evaluation is the combined positive score (CPS). In this case, the pathologist is supposed to use either the SP263 antibody (DAKO) or the 22C3 (DAKO) antibody. The methodology of scoring takes into account immune cells and tumor cells compared to the nonstained cells. The advised formula looks complicated, but pathologists are used to this algorithm. lt has been shown that if the CPS is greater than 10, pembrolizumab, which is another ICI, can be given [6]. The third score, which can be used in urothelial carcinoma is the Tumor Proportion Score (TPS), were only tumor cells are taken into account. Depending on the type of treatment, in bladder, this would be a treatment of maintenance in the adjuvant setting, more than 1% of tumor cells shall be stained with PD-L1 in order to receive nivolumab [7].

One of the major problems of testing is that different histological subtypes and divergent differentiations can express PD-L1 in a different manner. Squamous cell carcinoma has a tendency to express PD-L1 in a stronger way than the classical urothelial carcinoma [8]. The European Guidelines (EAU Guidelines) recommend PD-L1 expression in immunohistochemistry either on tumor cells or on immune cells in locally advanced high-risk metastatic urothelial carcinoma [9].

When comparing the different antibodies, it has clearly been shown that SP263 and 22C3 display consistent results, on the other hand, when comparing IHC to fluorescent in-situ hybridization (FISH), the results were not concordant [10].

Other strategies of testing targets are f.i. staining for Nectin-4. Recently, some authors have published, that based on IHC, Nectin-4 was less expressed in the metastatic setting, which was suggested to be problematic for the treatment with targeted therapies against Nectin-4 (EV-Pembro) [11]. Other recent articles show that Nectin-4 amplification can also be tested by FISH, which probably is a more precise technique in this setting [12]. Once again, it has to be underlined that Nectine-4 is not expressed the same in all subtypes. Nectine-4 is very well expressed from an immunohistochemical point of view in squamous cell carcinomas but not at all in sarcomatoid carcinomas. The same is true when looking at the mRNA expression for Nectine-4. Poor expressions exist in sarcomatoid and small cell bladder cancers [13].

Another immune histochemical test is TROP2, which is globally well expressed in tumors, but also in a strong way in normal urothelium. Another test, which seems to be promising, is the exploration of the ERBB2 pathway via HER-2, which is currently tested in several organs [14]. HER2 in bladder cancer seems to be an interesting target and there are specific therapies such as deruxtecan trastuzumab, which seem to be promising [10]. There is no official standard at the very moment on how to give an interpretation of HER-2 in bladder cancer, contrary to other organs (gastric and breast) [15,16]. In case of HER-2 2+ staining, additional in-situ hybridization is necessary at the very moment.

A recent article showed spatial heterogeneity between the tumor center, front and metastasis (as well lymph node as distant metastasis), which challenges the interpretation of all these markers in IHC [17].

An interesting approach of testing genetic alterations is the evaluation of FGFR alterations, especially FGFR3 mutations, which occur in around 40% of the luminal papillary tumors. Tumors with this kind of alteration can sometimes already be recognized on a histological slide as they often represent a specific subtype. Most of the large nested urothelial carcinoma and some of the plasmacytoid urothelial carcinoma have been reported to display FGFR3 mutations, although different molecular pathways have been described in these entities [18,19]. It must be underlined that the tumor is changing its molecular underpinnings when becoming invasive, in earlier stages [nonmuscle invasive bladder cancer (NMIBC)], FGFR alterations are detected more often (in up to 75%) than in the muscle invasive setting [20^▪▪^]. In the MIBC setting, luminal papillary tumors can display FGFR alterations in around 40% of cases. The advantage of testing mUC (metastatic) or LaUC (locally advanced) on a genetic level is, that heterogeneous expressions do not exist, which can be seen on the protein level of the tumor [21]. FGFR testing can be done in different manners, it is important to respect the preanalytical phase, which shall be done by a pathologist. Tumor material must be available in a sufficient amount in order to get good results, the best way of testing is next-generation sequencing (NGS), other techniques such as reverse transcriptase-polymerase chain reaction (RT-PCR) or FISH can also be done, but not all the existing alterations will be detected [22].

With regards to genetic testing, at the moment, the best known target is FGFR-3, which is a well known mutation now [23^▪▪^]. We must be aware that in MIBC, less of these alterations are seen than in the nonmuscle invasive setting. In Ta tumors, 80% display point mutation or upregulated expression. In T2–T4 tumors; only 10–15% display point mutation and around 40% of MIBC show an upregulated expression [1]. In FGFR hotspot mutations, like S249C, targeted therapies exist for this kind of mutated patients. Testing can be done either on the DNA or RNA level, the preanalytic phase is obviously important – and should to be done in a pathology lab. According to the technique 50–100 ng of DNA/RNA are needed, especially in amplicon-based or hybrid capture-based assays when doing Archer panel more RNA (100 ng) is requested. When doing FISH analysis, not every alteration will be detected, especially point mutations and fusions will not be seen. The same is true for PCR analysis where only some variants, fusions and point mutations can be detected. It is true that the turnaround time for FISH and PCR are shorter than for NGS, but the results are less robust. Failure rates can obviously exist, especially if not enough material is tested, the material is too old, and also sometimes noise mutations can occur. Most of the time in bladder cancer, the problem of too few materials is not a major issue. Several new approaches have been taken, also dealing with the nonmuscle invasive setting, f.i. trials like TAR-210 could maybe be interesting with regards to tailor treatments for these patients, which are seen much more often than patients with MIBC [24^▪^].

## KIDNEY CANCER

Kidney cancer accounts for 2.2% of all cancer cases, and in recent years, there has been an increasing number of cases of renal cell carcinoma (RCC). The classification has become complicated as more than 50 entities have been described and shall be recognized by the pathologist [25]. The increasing number of new cases might be linked to the environment but could also be linked to better imaging. Pathological prognostic models such as the tumor nodule metastasis staging, the international society of uropathology (ISUP) Grade and the presence of necrosis play an important role as well as the histological subtype. Some biomarkers have been evaluated: one of the markers is the ratio of neutrophil to lymphocytes in the blood, which has been considered as potential circulating biomarker and elevated neutrophil to lymphocyte ratio have been associated with poor prognosis. Another biomarker is the platelet to lymphocyte ratio (PLR) that has not proven its importance in kidney cancer. A newer biomarker is the systemic immune inflammation index (SIII) as well as the systemic inflammation response index (SIRI) [26]. A recent study could compare ISUP classification in new markers and prognosis, allowing a predicting model for a 2-year survival. This study could show that ISUP Grading is correlated with serum albumin levels [27]. Another recently describer marker is serum KIM-1 (kidney injury molecule-1), which could be a potential circulating biomarker in renal cell carcinoma [28]. KIM-1 is probably an interesting biomarker in case of minimal residual disease in RCC as well as in the case of KIM-1 high patients, the enrichment for the benefit of adjuvant immunotherapy does exist. A recent international group could show the importance of KIM-1, suggesting that elevated postnephrectomy plasma KIM-1 levels kinetics are prognostic; they think that KIM-1 is a biomarker for minimal residual disease in RCC. Patients with an increased KIM-1 during follow-up had worse disease-free survival, but in case of treatment with adalimubab, they had lower disease-free survival when associated with increased baseline expression of T-factor and TH1 signatures [28]. Another recent study could show that many blood-based biomarkers exist, mostly associated with worse outcomes such as inflammatory scores, circulating tumor-derived biomarkers (such as high IV Micro RNA 155-3p, high baseline CT DNA, high baseline increased CTCS urine treatment) but also metabolomics markers and soluplus factors such as BAFF, CXCL 13, WIGFA, SWIGFR 2 and IL-6, IL-8. Last but not least circulating immune cells such as BDL-1, CD4, CD8T and B-cells [29].

A recent study could also display interesting results in papillary renal cell carcinomas, which are less frequent than clear renal cell carcinomas. These patients might be treated with creatinkinase and ICIs, therefore the expression of PDL-1, PD-1 is obviously interesting. PDL-1-positive patients had, in this study, significantly shorter survival and multivariate analysis confirmed significant association between PDL-1 expression and shorter overall survival (*P* = 0,01) [30].

Nevertheless, it is important to mention that except KIM-1, which might be interesting but is still controversial, and PDL-1, no globally accepted biomarkers exist for RCCs, neither on tissue than blood based.

## PROSTATE

Prostate cancer (PCa) is the most frequent cancer amongst men but the lethality is less dramatic. PCa has become a tumor with many facettes [31].

Phosphatase and tensin homolog (PTEN) is a molecule which seems to play an important role. Alterations in this tumor suppressor gene are known for a long time and are linked to an aggressive PCa [32], IHC has been one of the major tools for a long time in the exploration of PTEN. New trials have been designed and are under review. A recent multiinstitutional trial from Germany showed a surprisingly high interobserver variability when giving an interpretation of the results [33]. A new way of testing might be the evaluation of the *PTEN* mRNA expression in mHSPC [34].

Approximately 25% of patients with metastatic PCa (mPCa) have homologous combination repair pathway gene mutations homologous recombination repair (HRR). Amongst those BRCA 1/2, especially BRCA 2- ATM, CDK12, RAD51C and PALB 2, play probably a major role [35]. One of the major challenges during these last years to choose whom to test as targeted therapies are available. At the very moment, recommendations for HRR testing exist in the case of metastatic castration-resistant PCa (mCRPC) ([36]). Different guidelines recommend different testing strategies. The EAU Guidelines suggest early PSA screening in men aged over 45, who have a family history of PCa or in BRCA carriers older than 40 years. Furthermore, it is recommended to do an early genetic sequencing of either the primary tumor or a tumor biopsy or a biopsy of the metastasis as soon as mCRPC exists. Many voices have risen during this last year, claiming that the right moment to test might be the metastatic hormone-sensitive prostate cancer (mHSPC), but as soon as a patient is metastatic or in progression, this test should be done [37]. Reflex testing at the very moment is not recommended. PARP inhibitors (PARPi) in the setting of mCRPC with HRR alterations are the treatment of choice, although not all HRR alterations have the same benefit of this targeted treatment – probably the best targets are homozygous BRCA 2 mutations and loss of the biallelic PALB2 [38]. Furthermore, there is always the questions between somatic and germline testing. Somatic testing is important for treatment decision and personalized medicine; on the other hand, germline testing is important in order to find familiar predisposition and inherited alterations. Once again, testing is most of the time in the hands of pathologists and should initially be done on the tissue – probably the most recent tissue is the best [37]. It is important to do good preanalytic screening (relatively fresh material – at least freshly cut materials). New adjuvant chemotherapy does not seem to change the results, in case of bone metastasis, the processing gets more complicated. The preanalytics is for the primary and metastatic site like the bladder (see above).

Liquid biopsies might provide an accurate view of the tumor's mutational status at the moment of the test, but we lack official recommendations of pathology societies. It is also important to mention that some older patients have clonal hematopoiesis of indeterminate potential (CHIPs) can exist, and give wrong results. Other technical challenges obviously exist for liquid biopsy techniques; therefore, they should be used with caution.

## TESTICULAR CANCER

An article by Lobo *et al.* is dedicated to testing residual disease and follow-up in testicular cancer, please refer to this article.

## CONCLUSION

Biomarker become an important tool in treatment decisions and follow-up of patients with urological malignancy. Many biomarkers exist, only few have found their way into daily practice, and many data lack validations. Nevertheless, we have made major progress during these last years, and the future of testing is not only bright but promising.

## Acknowledgements

*None*.

### Financial support and sponsorship

*None.*

### Conflicts of interest

*There are no conflicts of interest.*

## REFERENCES AND RECOMMENDED READING

Papers of particular interest, published within the annual period of review, have been highlighted as:

▪ of special interest
▪▪ of outstanding interest
